# The Dutch Intracerebral Haemorrhage Surgery Trial: study protocol for a randomised clinical trial of minimally invasive endoscopy-guided surgery in patients with spontaneous, supratentorial intracerebral haemorrhage

**DOI:** 10.1093/esj/aakaf008

**Published:** 2026-01-01

**Authors:** Floor N H Wilting, Axel Wolsink, Nadia H C Colmer, Floris H B M Schreuder, H Bart Brouwers, Hieronymus D Boogaarts, Diederik W J Dippel, Gerjon Hannink, Wilmar M T Jolink, Dagmar Verbaan, Marieke J H Wermer, Ruben Dammers, Catharina J M Klijn

**Affiliations:** Department of Neurology, Donders Institute for Brain, Cognition and Behaviour, Radboud University Medical Center, Nijmegen, the Netherlands; Department of Neurology, Donders Institute for Brain, Cognition and Behaviour, Radboud University Medical Center, Nijmegen, the Netherlands; Department of Neurosurgery, Erasmus University Medical Center, Erasmus MC Stroke Center & Center for Complex Microvascular Surgery, Rotterdam, the Netherlands; Department of Neurology, Donders Institute for Brain, Cognition and Behaviour, Radboud University Medical Center, Nijmegen, the Netherlands; Department of Neurosurgery, Elisabeth-TweeSteden Hospital, Tilburg, The Netherlands; Department of Neurosurgery, Radboud University Medical Center, Nijmegen, the Netherlands; Department of Neurology, Erasmus University Medical Center, Erasmus MC Stroke Center, Rotterdam, the Netherlands; Department of Medical Imaging, Radboud University Medical Center, Nijmegen, the Netherlands; Department of Neurology, Isala Hospital, Zwolle, the Netherlands; Department of Neurosurgery, Amsterdam University Medical Center, University of Amsterdam, Amsterdam, the Netherlands; Amsterdam Neuroscience, Neurovascular Disorders, Amsterdam, the Netherlands; Department of Neurology, University Medical Center Groningen, Groningen, the Netherlands; Department of Neurosurgery, Erasmus University Medical Center, Erasmus MC Stroke Center & Center for Complex Microvascular Surgery, Rotterdam, the Netherlands; Department of Neurology, Donders Institute for Brain, Cognition and Behaviour, Radboud University Medical Center, Nijmegen, the Netherlands

**Keywords:** cost-effectiveness, endoscopy-guided surgery, functional outcome, health economic evaluation, intracerebral haemorrhage, minimally invasive surgery, protocol, randomised controlled trial

## Abstract

**Background:**

Growing evidence suggests that surgical treatment of ICH may be beneficial, particularly when performed early, with minimally invasive procedures, and in patients with lobar ICH. However, the available evidence is limited by risk of bias, heterogeneity and imprecision, and data supporting a beneficial effect in deep ICH is limited.

**Aim:**

To determine whether early minimally invasive endoscopy-guided surgery in addition to standard medical management improves functional outcome in patients with spontaneous supratentorial ICH, compared with standard medical management alone.

**Study design:**

The Dutch ICH Surgery Trial (DIST) is a multicentre, prospective, randomised trial with open-label treatment and blinded end-point assessment conducted in 11 neurosurgical centres in the Netherlands. Six hundred adult patients with spontaneous supratentorial ICH with a haematoma volume ≥ 10 mL and an NIHSS score ≥2 will be enrolled. Patients will be randomised (1:1) to minimally invasive endoscopy-guided surgery within 8 hours of symptom onset in addition to standard medical management, or to standard medical management alone.

**Study endpoints:**

The primary outcome is the mRS score at 180 days. Secondary outcomes include the mRS at 90 and 365 days, safety and technical efficacy outcomes, quality-of-life measures and health economic evaluations up to 365 days. In addition, DIST will investigate blood and imaging biomarkers of secondary brain injury.

**Summary:**

Dutch ICH Surgery Trial assesses the efficacy of early endoscopy-guided surgery for patients with supratentorial ICH. Recruitment started in November 2022; as of October 2025, 235 participants have been enrolled. Completion of recruitment is expected in 2027.

**Trial registration:**

ClinicalTrials.gov NCT05460793.

## Graphical Abstract

**Graphical abstract:**
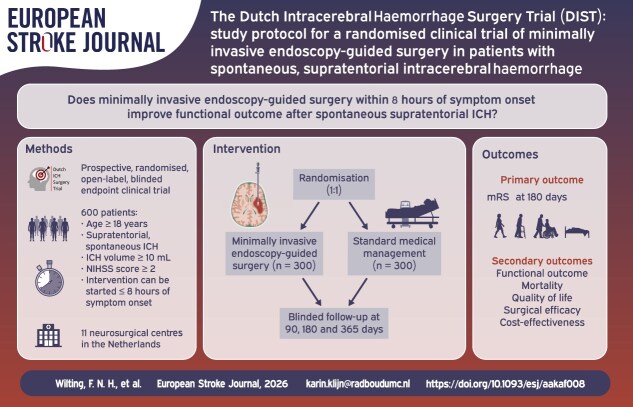

## Introduction

Intracerebral haemorrhage accounts for approximately 28% of all strokes worldwide and carries devastating consequences.^1^ One-month case fatality is 36%, and less than one-third of patients have a good functional outcome.^2^ Currently, treatments of proven benefit are limited to stroke unit care^3^ and early treatment of hypertension,^4^ preferably as part of an acute care bundle combining blood pressure lowering with anticoagulation reversal, access to critical care and neurosurgery in selected patients.^5-7^

The role of surgery in the treatment of ICH remains uncertain.^8^ The randomised trials STICH and STICH II showed no beneficial effect of surgical clot removal, mostly performed via craniotomy.^9^^,^^10^ However, in both studies, surgery was performed late after symptom onset (median times 30 and 26 hours) and over 20% of patients assigned to initial conservative treatment underwent surgery after secondary deterioration. SWITCH provided weak evidence suggesting that decompressive craniectomy without haematoma evacuation may benefit patients with severe deep ICH, but at the expense of severe disability among survivors.^11^ Regarding minimally invasive techniques, MISTIE III showed that catheter-based ICH aspiration with local administration of alteplase, started at a median of 59 hours after symptom onset, failed to improve functional outcome.^12^ More recently, ENRICH demonstrated that minimally invasive trans-sulcal parafascicular haematoma evacuation, performed at a median of 17 hours after last known well, improved functional outcome when compared to standard medical management.^13^ However, as only 2.6% of screened patients were included, and the effect was mainly attributable to patients with lobar ICH, more data are needed to confirm whether these results are robust and generalisable. The recently published MIND trial was terminated early following the ENRICH results and consequently lacked power to detect differences in functional outcome.^14^ Nevertheless, MIND demonstrated that minimally invasive endoscopy-guided surgery can effectively reduce ICH volume, with no apparent difference in efficacy between lobar and deep ICH, thereby supporting further randomised evaluation of this technique.

Surgical haematoma evacuation aims to alleviate the direct mass effect caused by the haematoma and to reduce intracranial pressure. In addition, haematoma evacuation may prevent secondary deterioration due to haematoma growth and attenuate secondary injury caused by blood degradation products.^15^ As haematoma expansion mostly occurs within the first hours after ICH,^16^ and the secondary injury cascade starts within hours after onset,^17^ early surgical intervention may offer advantages over later treatment.^18^ Moreover, minimally invasive techniques may mitigate the risks associated with open surgery, which may be particularly important for deep ICH. In the Dutch ICH Surgery Trial (DIST) pilot study, we demonstrated that minimally invasive endoscopy-guided surgery, performed within 8 hours of symptom onset, was safe, feasible and effectively reduced haematoma volume.^19^ Safety, logistical and technical feasibility have also been demonstrated in another study.^20^

The DIST aims to assess whether early minimally invasive endoscopy-guided surgery, in addition to standard medical management, improves functional outcome after spontaneous supratentorial ICH when compared to standard medical management alone.

**Table 1:** Inclusion and exclusion criteria

**Inclusion criteria**	**Exclusion criteria**
Age ≥ 18 years NIHSS score ≥ 2 Supratentorial non-traumatic ICH confirmed by CT, without a confirmed causative vascular lesion on admission CTA (eg, aneurysm, arteriovenous malformation, dural arteriovenous fistula, cerebral venous sinus thrombosis), or other known underlying lesion (eg, tumour, cavernoma) Haematoma volume ≥ 10 mL Intervention can be started ≤ 8 hours of symptom onset Written informed consent (deferred)	Pre-stroke mRS score ≥ 3 ICH-GS score ≥ 11^31^ Haemorrhage due to haemorrhagic transformation of an infarct Untreated coagulation abnormalities, including INR > 1.3, treatment with heparin and treatment with factor Xa inhibitors. Patients on vitamin K antagonists can be included after correction of the INR, and patients on dabigatran can be included after reversal with idarucizumab Moribund (eg, coning, bilateral dilated unresponsive pupils), or progressively deteriorating clinical course with imminent death Pregnancy DIST-INFLAME substudy: patients who use immunosuppressive or immune-modulating medication

Abbreviations: ICH-GS = Intracerebral Haemorrhage-Grading Scale; INR = international normalised ratio.

## Methods

### Study design

Dutch ICH Surgery Trial (www.dutch-ich.nl) is a multicentre, prospective, randomised (1:1) controlled trial with open-label treatment and blinded endpoint assessment, investigating whether minimally invasive endoscopy-guided surgery within 8 hours of symptom onset, in addition to standard medical management, improves functional outcome at 6 months, in comparison with standard medical management alone. The trial will be conducted in 11 neurosurgical centres in the Netherlands (Supplemental material).

In parallel, DIST-ABC (A Budget Impact and Cost-effectiveness analysis) will evaluate the cost-effectiveness and budget-impact of minimally invasive endoscopy-guided surgery from a healthcare system perspective.

The DIST-INFLAME substudy will: (1) assess whether patients treated with minimally invasive surgery develop less perihaematomal oedema than patients treated with standard medical management alone, (2) assess whether this effect is modified by the CTP permeability surface-area product around the ICH at baseline, and (3) compare immune profiles over time in venous blood between surgically treated patients and patients treated with standard medical management alone.

DIST is embedded within the Collaboration for New Treatments of Acute Stroke (CONTRAST 2.0) consortium (www.contrast-consortium.nl), which supports multicentre stroke research in the Netherlands. The study will be conducted according to the principles of the Declaration of Helsinki and in accordance with the Medical Research Involving Human Subjects Act Wet medisch-wetenschappelijk onderzoek met mensen (WMO). The study has been approved by the Erasmus MC medical ethics review committee (NL80112.078.22) and is registered on ClinicalTrials.gov (NCT05460793).

### Patient population

Adults with a spontaneous, supratentorial ICH presenting to 1 of 11 neurosurgical centres, or referred to these centres by regional hospitals, can be enrolled. Inclusion and exclusion criteria are listed in Table 1.

### Randomisation

Eligible patients will be randomised by local site personnel via a secure web-based internet system. Patients will be allocated in a 1:1 ratio to either minimally invasive endoscopy-guided surgery in addition to standard medical management (intervention group), or to standard medical management alone (control group), using permuted blocks with random block sizes of 10 or 20 stratified by neurosurgical centre. It will not be possible to view the treatment allocation before the patient is randomised. Due to the nature of the intervention, both patient and local site personnel cannot be blinded for the assigned treatment.

### Deferred consent procedure

Patients will be enrolled using a deferred consent procedure in accordance with the Medical Research Involving Human Subjects Act (WMO).^21^ Written informed consent to continue participation in the study will be obtained from the patient or their representative by trained research personnel, as early as deemed appropriate and reasonable after randomisation (control group) or intervention (intervention group). In patients allocated to the intervention group, we will ask the patient or their representative for consent for the surgical treatment according to common clinical practice for any procedure. Patients who have indicated, as part of advanced care planning, that they do not want to undergo any surgical treatment will not be enrolled. When a representative has provided consent and the patient regains decision-making capacity, we will ask the patient for written consent at that time. If a patient has died before deferred consent has been obtained, the legal representative will be informed about trial participation. Patients or representatives who do not consent will be asked for permission to use routinely collected data and materials. If patients or their representatives object to the use of their data, their data will be registered in an anonymized registry to obtain data on in-hospital rebleeding occurrence and in-hospital mortality for the purpose of safety analyses. All other study information will be completely erased from the patient’s study record.

### Intervention

Patients allocated to the intervention group will undergo minimally invasive endoscopy-guided surgery within 8 hours of symptom onset. The trial allows the use of any Conformité Européenne (CE)-marked minimally invasive neuronavigation integrated endoscopy-guided device; currently, this includes only the Artemis™ Neuro Evacuation Device (Penumbra, Inc.). With the neuronavigation software available at the neurosurgical centre, a trajectory is selected that is considered technically feasible, safe and allows access to the longest axis of the haematoma. Intracranial access is obtained via a burr hole, after which a peel-away sheath is introduced to facilitate safe passage of the endoscope during the procedure. Surgery is performed by credentialed neurosurgeons in accordance with the DIST surgical protocol. Credentialing includes successful participation in the DIST pilot study or completion of a dedicated qualification process. The DIST surgical protocol and credentialing procedure are outlined in the Supplemental material.

Both patients in the intervention and control groups will receive standard medical management according to the Dutch^22^ and European Stroke Organisation^7^^,^^23^ guidelines for the management of spontaneous ICH.

### Study procedures

**Figure 1:**
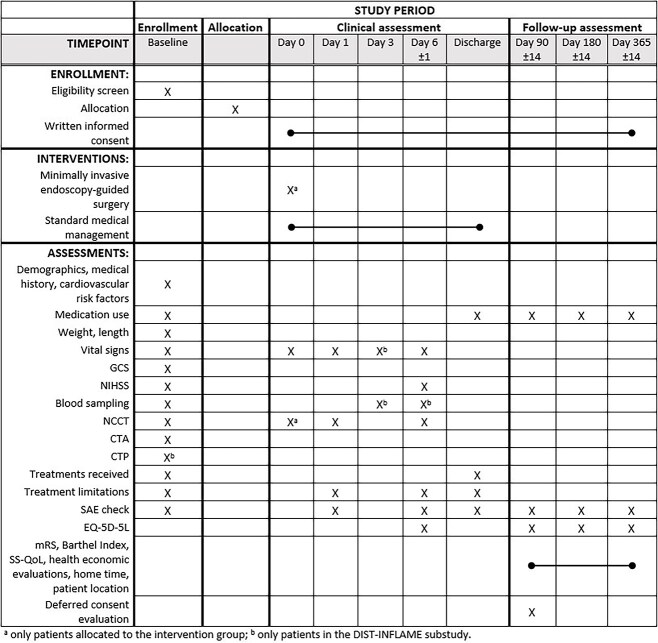
SPIRIT figure of Dutch ICH Surgery Trial (DIST). Abbreviations: EQ-5D-5L = EuroQol five dimensions-five levels; GCS = Glasgow coma scale; NCCT = non-contrast computed tomography; SAE = serious adverse event; SS-QoL = Stroke Specific Quality of Life.

In addition to the study intervention, patients will undergo study assessments at day 0 (admission), day 1 (±6 hours), day 3 (±12 hours), day 6 (±1 day, or discharge if earlier) and during follow-up at days 90, 180 and 365 (±14 days) (Figure 1).

#### Clinical data

Baseline characteristics will be assessed by treating physicians upon presentation to the emergency department. Vital signs will be collected at 1, 6, 12 and 24 hours post-admission, day 3 (±12 hours; DIST-INFLAME only) and day 6 (±1 day). NIHSS and EuroQol five-dimension five-level (EQ-5D-5L) assessments will be performed at day 6 (±1 day). Details of the surgical intervention, treatment limitations, interventions, diagnoses and medication use will also be recorded during hospitalisation.

#### Blood sampling

We will collect blood at baseline obtained as part of routine clinical practice. We will draw additional blood samples (serum, plasma EDTA, whole blood EDTA, citrate and PAXgene tubes) in all patients at baseline, and in patients in the DIST-INFLAME substudy at day 3 (±12 hours) and day 6 (±1 day).

#### Neuroimaging

Patients will undergo a baseline brain non-contrast CT (NCCT) and CTA. To assess eligibility for the study, haematoma volume will be calculated using the ABC/2 formula^24^ or volumetric 3D software upon presentation at the emergency department. In patients in the DIST-INFLAME substudy, a baseline CTP will be performed according to a prespecified protocol. An immediate postoperative NCCT will be performed to assess ICH evacuation. In addition, all patients will undergo NCCT at 24 hours (±6 hours) and day 6 (±1 day). The CT acquisition protocol requirements are described in the Supplemental material.

#### Follow-up assessment

Trained research personnel, unaware of treatment allocation, will assess the mRS, Barthel Index, EQ-5D-5L and Stroke-Specific Quality of Life (SS-QoL) scale using standardised reports via telephone interviews at 90, 180 and 365 days. Similarly, home time and patient location will be assessed at these time points. Health economic evaluations will be evaluated using questionnaires based on the iMTA Medical Consumption Questionnaire (iMCQ), the iMTA Productivity Cost Questionnaire (iPCQ), and the iMTA Valuation of Informal Care Questionnaire (iVICQ).

#### Deferred consent evaluation

In a sample of 100 patients, we will evaluate the acceptability of the deferred procedure by means of a pre-piloted questionnaire between 90 and 180 days. In addition, a subsample of patients will be invited to an in-depth interview.

### Study outcomes

The primary outcome is the mRS score at 180 days (±14 days).^25^ The mRS is a 7-category ordinal disability scale with scores ranging from 0 (no symptoms at all) to 6 (death). Secondary, safety, technical efficacy and DIST-INFLAME substudy outcomes are listed in Table 2.

**Table 2:** Study outcomes in Dutch ICH Surgery Trial (DIST)

Primary endpoint mRS score at 180 days (ordinal distribution) Secondary endpoints mRS score at 90 and 365 days (ordinal distribution) Good outcome (mRS score 0-2) at 90, 180 and 365 days Favourable outcome (mRS score 0-3) at 90, 180 and 365 days All other possible dichotomisations of the mRS score at 90, 180 and 365 days NIHSS score at 6 days (or discharge if earlier) Death at 90, 180 and 365 days Barthel Index score at 90, 180 and 365 days Quality of life assessed with the EQ-5D-5L at 6 days (or discharge if earlier), and 90, 180 and 365 days Quality of life assessed with the SS-QoL at 90, 180 and 365 days Resource use assessed with the iMCQ and iPCQ at 90, 180 and 365 days Burden for the caregiver assessed with the iVICQ at 90, 180 and 365 days Home time at 90, 180 and 365 days Patient location over the first 90, 180 and 365 days Safety outcomes Death within 24 hours Procedure-related complications within 7 days Case-fatality at 7 and 30 days Technical efficacy outcomes Percentage volume reduction based on baseline NCCT and NCCT at 24 hours Percentage participants with haematoma volume reduction ≥70% Percentage participants with haematoma volume reduction ≥80% Percentage participants with remaining haematoma volume ≤ 10 mL Percentage participants with remaining haematoma volume ≤ 15 mL Percentage participants with conversion to craniotomy DIST-INFLAME substudy outcomes Perihaematomal oedema assessed on NCCT at 6 days (or discharge if earlier) mRS score at 180 days Immune and metabolomic profiles in venous blood assessed at 3 and 6 days (or discharge if earlier)

Abbreviations: EQ-5D-5L = EuroQol five dimensions-five levels; iMCQ = iMTA Medical Consumption Questionnaire; iPCQ = iMTA Productivity Cost Questionnaire; iVICQ = iMTA Valuation of Informal Care Questionnaire; NCCT = non-contrast computed tomography; SS-QoL = Stroke Specific Quality of Life.

Adjudication of the mRS score will be performed by an outcome assessment committee blinded to treatment allocation (Supplemental material). Their assessments will be based on the standardised reports of the follow-up interviews by trained research personnel. Information on follow-up assessments in the main study database will only be visible by outcome assessors and the independent, unblinded statistician of the data safety monitoring board (DSMB).

Imaging will be assessed by experienced neuroradiologists of the imaging assessment committee (Supplemental material). Baseline imaging will be evaluated blinded for baseline characteristics, treatment allocation and outcome measures. Follow-up imaging will be evaluated blinded for baseline characteristics, outcome measures and for the results of baseline imaging.

### Data and safety reporting

All serious adverse events (SAEs) occurring until the end of follow-up will be centrally assessed by the SAE adjudication committee (Supplemental material). Study safety will be monitored by an independent DSMB consisting of a neurologist, a neurosurgeon and an independent methodologist/statistician (Supplemental material). Interim safety and efficacy analyses will be performed at least annually or after completion of 30-day follow-up of the first 50 and subsequent 100, 250 and 400 enrolled patients. Results of interim analyses will be provided to the DSMB by an independent, unblinded statistician in strict confidence, along with any other analyses the DSMB may request. The steering committee will remain blinded to the results of interim analyses of efficacy and safety.

### Statistical analysis

The primary analyses will be performed according to the intention-to-treat principle. Baseline data by treatment allocation will be reported with standard statistical procedures. Missing values for baseline characteristics and outcomes will be reported. For the analyses, missing baseline characteristics and outcomes will be imputed using multiple imputation.

The primary effect parameter will be reported as a common odds ratio, estimated with ordinal logistic regression, which represents the shift across the 7-category mRS measured at 180 days. The treatment effect estimate will be adjusted for neurosurgical centre and known prognostic variables: age, pre-stroke mRS, time from symptom onset to randomisation, systolic blood pressure on admission, stroke severity (NIHSS), ICH volume, presence of intraventricular haemorrhage and antiplatelet or oral anticoagulant use immediately before stroke onset. Adjusted and unadjusted estimates with corresponding 95% CIs will be reported. The adjusted analysis with multiple imputation will be considered the primary analysis. In addition, we will perform a per-protocol and as-treated analysis.

Secondary effect parameters will be analysed using linear, logistic or ordinal regression analyses as appropriate, applying the same adjustment variables as for the primary outcome.

The effect of the intervention on the mRS will be analysed in pre-specified subgroups determined by the following variables: tertiles of age; sex; location of ICH (deep vs lobar); tertiles of systolic blood pressure at baseline; tertiles of NIHSS at baseline; tertiles of ICH volume; tertiles of time from symptom onset to randomisation; presence of CTA spot sign and prior use of antiplatelet agents or oral anticoagulants.

For the cost-effectiveness analysis, costs and quality-adjusted life years will be calculated in both groups over the 12-month follow-up period. We will perform a model-based economic evaluation to estimate the lifetime cost-effectiveness of the intervention.

Further details will be provided in a separate statistical analysis plan.

### Sample size calculation

Sample size estimations were based on the distribution of outcomes in MISTIE III,^12^ and on data from the DIST pilot study.^19^ We assumed a distribution of mRS in controls as follows: mRS 0: 0%; mRS 1: 5%; mRS 2: 15%; mRS 3: 20%; mRS 4: 25%; mRS 5: 15%; mRS 6: 20%, and a favourable treatment effect with a common odds ratio of 1.49, corresponding to an absolute risk difference of mRS 0-3 of 11%. In a Monte Carlo simulation with 5000 runs, we computed the proportion of positive trials for a given sample size. This yielded a sample size of 800, providing a 90% power to detect a true treatment effect, with a 2-sided alpha of 0.05. In the analysis, we will use covariate adjustment, under the assumption that this reduces the sample size by 25%.^26^ Therefore, the final sample size will consist of 600 patients, 300 for each treatment group.

### Trial status

Patient recruitment started in November 2022 and is expected to finish in July 2027. Status of enrolment can be followed at www.dutch-ich.nl (October 2025: *n* = 235).

## Discussion

DIST is a multicentre randomised trial designed to evaluate whether minimally invasive endoscopy-guided surgery performed within 8 hours of symptom onset, in addition to standard medical management, improves functional outcome after 6 months, compared to standard medical management alone in patients with spontaneous supratentorial ICH. Secondary outcomes include safety, technical efficacy, quality of life and cost-effectiveness.

In the recently updated Cochrane review on surgery for spontaneous supratentorial ICH, low-certainty evidence suggested that minimally invasive surgery may increase the chance of good functional outcome.^8^ The effect on mortality was supported by moderate-certainty evidence, whereas the effect on health-related QoL was very uncertain.^8^ Most included studies had high risk of bias and pooled estimates were often imprecise, underscoring the need for high-quality, adequately powered studies to guide clinical practice.

In addition to DIST, several other ongoing randomised trials aim to address this evidence gap,^8^ including EMINENT-ICH (NCT05681988), EVACUATE (NCT04434807), NESICH (NCT05539859) and REACH (NCT06870812). These trials differ in inclusion criteria, timing of surgery and technique of the intervention.

In ENRICH and MISTIE III, only patients with haematoma volumes greater than 30 mL were included. Multiple ongoing studies have now adopted lower thresholds—10 mL in DIST, 20 mL in EVACUATE, EMINENT-ICH and REACH and 25 mL in NESICH—reflecting the hypothesis that minimally invasive surgery may also benefit patients with smaller haematomas, as accumulating evidence demonstrates that minimally invasive surgery can be performed safely. DIST also has broad inclusion criteria with regard to age and clinical condition, in contrast to some other studies, to increase generalisability.

Another distinguishing feature of DIST is the early time window for surgery—within 8 hours of symptom onset—shared only with EVACUATE. Other studies allow intervention up to 24–72 hours. Observational data suggested that haematoma removal may be most effective when performed early, with a 5% increase in the odds of achieving a good functional outcome for each hour surgery started earlier.^27^ Nonetheless, the optimal timing of surgery remains uncertain and warrants further investigation.

A potential advantage of endoscopy-guided evacuation over other minimally invasive approaches is that it permits direct visualisation of residual haematoma and management of active bleeding, including coagulation of focal bleeding points and management of diffuse bleeding through irrigation.^28^ This facilitates effective removal of the haematoma and may result in lower rebleeding rates compared to other minimally invasive approaches. Effective haematoma removal is critical, as multiple studies have demonstrated that lower residual volume is associated with better functional outcome.^29^^,^^30^ Therefore, the DIST protocol includes an NCCT immediately after surgery, with the option to return to the operating room in case of substantial residual haematoma. Currently, it is unclear whether 1 technique is superior to the other with regard to safety and effectiveness. In DIST, participating neurosurgeons must meet credentialing requirements and adhere to a detailed surgical protocol (Supplemental material) to optimise safety and minimise variability.

Completion of all ongoing studies is essential to provide evidence on whether, when, how and in which patients minimally invasive surgery may improve functional outcome. Ultimately, an individual patient data meta-analysis will provide a more granular understanding of treatment effect modifiers and inform personalised treatment strategies. A protocol for this meta-analysis is currently being developed.

In conclusion, DIST is designed to provide robust, high-quality evidence on the effect of minimally invasive endoscopy-guided surgery performed within 8 hours of symptom onset in patients with spontaneous supratentorial ICH. Its results will contribute much-needed new data on the efficacy of haematoma evacuation in this devastating disease, and may guide future clinical decision-making and trial design.

## Supplementary Material

aakaf008_DIST_Protocol_Paper_ESJ_Supplemental_material_d_d_20251016_edited

## Dutch ICH Surgery Trial Study Group, part of the CONTRAST consortium

Floor N.H. Wilting, Axel Wolsink, Nadia H.C. Colmer, Floris H.B.M. Schreuder, Saloua Akoudad, Renate M. Arntz, H. Bart Brouwers, Hieronymus D. Boogaarts, Jonathan M. Coutinho, J. Marc C. van Dijk, Diederik W.J. Dippel, Ellis S. van Etten, Roel H.L. Haeren, Gerjon Hannink, Ben P.W. Jansen, Paula M. Janssen, Wilmar M.T. Jolink, Kuan H. Kho, Wouter A. Moojen, Martin Podlogar, René Post, Inger R. de Ridder, Dagmar Verbaan, Marieke J.H. Wermer, Ido R. van den Wijngaard, H. Bart van der Worp, Albert van der Zwan, Ruben Dammers and Catharina J.M. Klijn.
