# Supplementary material for: The Dutch Intracerebral Haemorrhage Surgery Trial: study protocol for a randomised clinical trial of minimally invasive endoscopy-guided surgery in patients with spontaneous, supratentorial intracerebral haemorrhage
Source: Eur Stroke J. 2026 Jan 1;11(1):aakaf008. doi: 10.1093/esj/aakaf008 (PMC12866656; doi:10.1093/esj/aakaf008)
Supplement: aakaf008_DIST_Protocol_Paper_ESJ_Supplemental_material_d_d_20251016_edited [file aakaf008_dist_protocol_paper_esj_supplemental_material_d_d_20251016_edited.docx]

**Supplemental material to:**

**The Dutch Intracerebral Haemorrhage Surgery Trial: study protocol for a randomised clinical trial of minimally invasive endoscopy-guided surgery in patients with spontaneous, supratentorial intracerebral haemorrhage**

Floor N. H. Wilting, Axel Wolsink, Nadia H. C. Colmer, Floris H. B. M. Schreuder_,_ H. Bart Brouwers, Hieronymus D. Boogaarts, Diederik W. J. Dippel, Gerjon Hannink_,_ Wilmar M. T. Jolink, Dagmar Verbaan, Marieke J. H. Wermer, Ruben Dammers and Catharina J. M. Klijn, on behalf of the Dutch ICH Surgery Trial Study Group, part of the CONTRAST consortium.

**Table of contents**

[1. List of study committees 2](#_Toc207184515)

[2. Surgical protocol 3](#_Toc207184516)

[2.1. Investigational product 3](#_Toc207184517)

[2.2. Credentialing procedure 3](#_Toc207184518)

[2.3. Surgical protocol 3](#_Toc207184519)

[3. CT acquisition protocol and imaging requirements 6](#_Toc207184520)

[3.1. Clinical imaging in patients with ICH 6](#_Toc207184521)

[3.2. Imaging acquisition protocols DIST 6](#_Toc207184522)

[3.2.1. Non-contrast CT brain acquisition protocol requirements 7](#_Toc207184523)

[3.2.2. CT-angiography head(-neck) acquisition protocol requirements 8](#_Toc207184524)

[3.3.3. CT-perfusion brain acquisition protocol requirements 9](#_Toc207184525)

[4. References 10](#_Toc207184526)

# 1. List of study committees

Executive and writing committee

C.J.M. Klijn (Radboud University Medical Center, Nijmegen); R. Dammers (Erasmus University Medical Center, Rotterdam); F.H.B.M. Schreuder (Radboud University Medical Center, Nijmegen); H.D. Boogaarts (Radboud University Medical Center, Nijmegen); D. Verbaan (Amsterdam University Medical Center, Amsterdam); M.J.H. Wermer (University Medical Center Groningen, Groningen); D.W.J. Dippel (Erasmus University Medical Center, Rotterdam); H.B. Brouwers (Elisabeth Tweesteden Hospital, Tilburg); W.M.T. Jolink (Isala Hospital, Zwolle); F.N.H. Wilting (Radboud University Medical Center, Nijmegen); A. Wolsink (Radboud University Medical Center, Nijmegen); N.H.C. Colmer (Erasmus University Medical Center, Rotterdam).

Steering committee

- Radboud University Medical Center, Nijmegen: C.J.M. Klijn (principal investigator), F.H.B.M. Schreuder, H.D. Boogaarts, F.N.H. Wilting, A. Wolsink
- Erasmus University Medical Center, Rotterdam: R. Dammers (principal investigator), D.W.P. Dippel, P.M. Janssen, N.H.C. Colmer
- Amsterdam University Medical Center, Amsterdam: D. Verbaan, J.M. Coutinho, R. Post
- Elisabeth Tweesteden Hospital, Tilburg: H.B. Brouwers, B.P.W. Jansen
- Haaglanden Medical Center, Den Haag: W.A. Moojen, I.R. van den Wijngaard
- Isala Hospital, Zwolle: W.M.T. Jolink, M. Podlogar
- Leiden University Medical Center, Leiden: E.S. van Etten, W.A. Moojen
- Maastricht University Medical Center, Maastricht: I.R. de Ridder, R.H.L. Haeren
- Medisch Spectrum Twente, Enschede: R.M. Arntz, K.H. Kho
- University Medical Center Groningen, Groningen: M.J.H. Wermer, S. Akoudad, J.M.C. van Dijk
- University Medical Center Utrecht, Utrecht: A. van der Zwan, H.B. van der Worp

Imaging assessment committee

F.J.A. Meijer (Radboud University Medical Center, Nijmegen); S.A.H. Pegge (Radboud University Medical Center, Nijmegen); B.J. Emmer (Amsterdam University Medical Center, Amsterdam); A.A. Jacobi-Postma (Maastricht University Medical Center, Maastricht); S.D. Roosendaal (Amsterdam University Medical Center, Amsterdam); H. Ahmad (Erasmus University Medical Center, Rotterdam); J.W. Dankbaar (University Medical Center Utrecht, Utrecht); H.Z. Flach (Isala, Zwolle).

Outcome assessment committee

J.M. Coutinho (Amsterdam University Medical Center, Amsterdam); D.W.J. Dippel (Erasmus University Medical Center, Rotterdam); J. Staals (Maastricht University Medical Center, Maastricht); M. Uyttenboogaart (University Medical Center Groningen, Groningen).

Adverse event adjudication committee

J. Staals (Maastricht University Medical Center, Maastricht); R.J. van Oostenbrugge (Maastricht University Medical Center, Maastricht).

Data safety and monitoring board

C. Anderson (UNSW Sydney, Australia; Chair); H. Patel (University of Manchester, UK; Member); L. Billot (UNSW Sydney, Australia; Member); J.W. van Dalen (Amsterdam University Medical Centre, the Netherlands; Independent unblinded statistician).

Trial statistician and methodologist

G. Hannink (Radboud University Medical Center, Nijmegen).

# 2. Surgical protocol

This surgical protocol is supplemental to the Dutch ICH Surgery Trial (DIST). This study intends to study whether minimally invasive endoscopy-guided surgery for treatment of spontaneous supratentorial ICH, performed within eight hours of symptom onset, improves functional outcome at six months. To ensure minimal performance bias we outline a surgical protocol to which including centres are obliged to adhere.

## 2.1. Investigational product

The devices allowed into the trial are minimally invasive neuronavigation integrated endoscopy-guided devices. At present, this only includes the Artemis^TM^ Neuro Evacuation Device (Penumbra, Inc.), which is CE marked. The choice of a particular device is left to the discretion of the neurosurgeon. When other devices will become available, they may be used when they are deemed admissible by the steering committee.

## 2.2. Credentialing procedure

The study intervention is to be performed by credentialed neurosurgeons. Credentialing includes successful participation in the DIST pilot study or completion of the qualification procedure (run-in phase). The qualification procedure consists out of a detailed instructional training on the stereotactic-guided endoscopic procedure, including direct mentoring of the detailed step-by-step surgical protocol by the surgical principal investigator and hands-on training in a dry-lab setting. Additionally, neurosurgeons will at least complete three run-in cases as principal surgeon that satisfy the criteria for eligibility before becoming eligible to take the primary responsibility for the surgery on patients enrolled into the study. The run-in cases are reviewed and evaluated by the expert assessment team, consisting of three neurosurgeons proficient in the surgical procedure and cognizant of the logistical aspects and potential hazards and complications. When the three run-in cases meet the criteria, the team will confirm the qualification of the neurosurgeon to the executive committee. If not, the team will recommend that extra run-in cases will be performed and evaluated. Per participating neurosurgical centre, at least one neurosurgeon will have to finish the qualification process successfully to become eligible to start inclusion for DIST. The qualified surgeons, in turn, can teach other participating neurosurgeons at their neurosurgical centre to perform the surgery as well. For the latter to become eligible to take the primary responsibility for the surgery on patients included in DIST, they will need to complete three cases under the supervision of a qualified surgeon. When a qualified surgeon is present during the surgery, the patient can also be included in DIST.

## 2.3. Surgical protocol

All participants in DIST undergoing minimally invasive endoscopy-guided surgery will be treated according to this surgical protocol and the local institutional guidelines.

**Pre-operative neuroimaging and planning for frameless image-guided endoscopic surgery**

Depending on the institution and neuronavigation systems used, appropriately protocolled CT-imaging studies will be uploaded into the neuronavigation software for procedural planning and guidance. The use of surface merging or fiducial markers will be at the discretion of the surgeon. If an additional neuronavigation (non-contrast CT) scan is necessary, it will be performed as soon as possible after randomisation in the surgical arm. A trajectory will be selected that is both technically feasible and allows access to the longest possible axis of the haematoma. For this, we adhere to the protocol as described in the ICES study.^1^ The ideal trajectory, which is parallel to the longitudinal axis of the haematoma, is selected determining a candidate entry and target point. One of three approaches will be selected: (A) anterior frontal lobe approach, (B) posterior parietal lobe approach, or (C) surface cortical approach; each of which will be designed to be parallel and in the middle of the longitudinal axis of the haematoma while avoiding the internal capsule, vasculature, eloquent white matter tracts, and ventricles.

**Surgical procedure**

The patient is placed upon the procedural table according to the approach used. The procedure is performed under general anaesthesia, and prophylactic antibiotics are administered according to local protocol. An external localization array or other neuronavigation localization is placed for registration according to the neuronavigation system in use. Once the appropriate entry point is identified, this area is prepared and sterile draped according to institutional guidelines. The image guidance probe is positioned over the candidate entry point. The virtual extension of the probe tip can be employed to interrogate the candidate entry points to assess whether the endoscope sheath will transgress any critical functional areas. If need be, the entry point can be adapted intra-operatively.

Hereafter, a 1.5-2.0 cm burr hole or minicraniotomy (maximum diameter 3-5 cm) of a size large enough to accommodate the selected endoscopy sheath is created. The dura is opened and the cortical surface coagulated and incised. A localization array (e.g., Instrument Adapter Clamp with Instrument Adapter Array, Brainlab AG) is attached to the selected neuroendoscopic sheath and registered to the navigation system. Using neuronavigation, the sheath is then advanced into the targeted landing zone until the distal aspect is located 2/3 of the longitudinal axis of the haematoma (point #1), after which the inner obturator is removed. The sheath is then stabilized (e.g., manually stabilized, mechanically stabilized, or peeled away and stapled down) into position.

The neuroendoscope is then inserted into the sheath, and under direct visualization the Artemis^TM^ Neuro Evacuation Device is placed through the working channel of the trocar. The sheath is irrigated at the discretion of the surgeon using the irrigation port of the endoscope. Preferably, Lactated Ringer’s Irrigation or Sterofundin® is used as an irrigant (instead of Sodium Chloride Irrigation Solution). The irrigant is intermittently aspirated with the Artemis^TM^ system until a clear working view is created within the sheath that allows visualization of the surgical field at the sheath tip. The Artemis^TM^ wand is advanced under direct visualization to, or just beyond the tip of the sheath and actuated to evacuate the blood products. If the working view becomes obscured by blood products within the sheath, additional irrigation and aspiration is performed intermittently to clear the field. This is repeated until no further clot can be evacuated at this location. The endoscope sheath is then irrigated to be sure that there is no evidence of active bleeding. If active bleeding is detected, irrigation is continued until the bleeding stops. If the bleeding does not stop adequately, the endoscope is introduced into the sheath, fixed in place, after which the bleeding point identified endoscopically and coagulated. Once haemostasis is obtained, the endoscope sheath is retracted to approximately 1/3 of the longitudinal axis of the haematoma cavity (point #2). The suctioning and irrigation process is then repeated at point #2. Suctioning is continued until at least 75% of the haematoma volume is thought to be removed, though maximal haematoma evacuation is desirable. Lastly, the endoscope is reintroduced to ensure there is no sign of active bleeding that may require additional irrigation or bipolar coagulation. However, no rotational steering of the sheath or lateral exploration of the haematoma cavity is permitted. Subsequently, the endoscope and sheath are removed. These endoscopic techniques are elaborately described elsewhere as well.^2,3^ The cortical surface is carefully inspected to ensure that there is no bleeding from the corticotomy. Finally, the dura and skin are closed routinely.

A control NCCT is performed immediately after surgery, or intra-operatively if possible (hybrid room with intra-operative CT), to confirm adequate haematoma evacuation and to assess for any complications (e.g., rebleeding, hydrocephalus, increased mass effect). The surgical goal is to reduce the haematoma volume by at least 75%. It is at the surgeon’s discretion to opt for an immediate return to the operating room to evacuate any residual haematoma.

**Postoperative care protocol**

Patients are either admitted to the (neuro-)intensive care unit (ICU) or a dedicated stroke unit for postoperative care. Neurological evaluation is performed according to institutional guidelines. Hypertension is treated according to National Guidelines as part of standard medical management, as is the case with patients in the non-surgical arm of the study. The aim is to achieve a target systolic blood pressure of 140 mmHg, if necessary, using intravenous hypertensive agents in the acute phase.

Ideally, patients should emerge rapidly from anaesthesia to permit immediate assessment of surgery results and to provide a baseline for continued postoperative neurologic follow-up. Nevertheless, there are some categories of patients in whom early awakening will not be deemed appropriate by the attending neurosurgeon (e.g. preoperative impaired consciousness or inadequate airway control, high postoperative risk of brain oedema, elevated ICP, or deranged intracerebral haemostasis). This will remain at the discretion of the surgeon.

Prophylactic use of low-molecular-weight heparin (LMWH) in immobile patients is allowed at least 48 hours after the onset of the intracerebral haemorrhage. Intermittent pneumatic compression and elastic stockings can be applied in the first 72 hours.

Restarting anticoagulant or antiplatelet medication in patients with a clear indication will be allowed as of three days after surgery. There are no trials to determine the optimal timing of restarting anticoagulants after ICH. The decision on whether and when to restart this medication is left to the local team and will depend on the indication for the antithrombotic treatment and a careful risk/benefit assessment.

# 3. CT acquisition protocol and imaging requirements

## 3.1. Clinical imaging in patients with ICH

**Non-contrast CT and CT angiography**

Patients suspected of an acute stroke and no significant renal insufficiency or contrast allergy routinely undergo a stroke CT study on presentation at the emergency department, which consists of a non-contrast CT (NCCT) of the brain and a CT-angiogram (CTA) of the (cervical) and intracranial arteries.

Before randomisation in DIST, an NCCT and CTA should be performed to assess eligibility for the study. In addition, 24 hours (± 6 hours) after randomisation, and 6 ± 1 day after randomisation (or at discharge if earlier) a NCCT should be performed to assess the haematoma volume and perihaematomal oedema. Patients in the intervention group will undergo an additional NCCT for the purpose of neuronavigation if deemed necessary by the operating neurosurgeon, and an NCCT immediately after surgery to assess the achieved reduction in ICH volume.

**CT perfusion**

In some sites, a CT perfusion (CTP) is already performed in patients with an intracerebral haemorrhage, as a standard part of the stroke CT study in addition to the NCCT and CTA upon presentation to the emergency department. The standard CTP acquisition protocol of these sites consists of one phase with a short acquisition time, which is used to calculate the perfusion parameters. However, for permeability measurements, which are relevant for assessment of blood-brain barrier (BBB) breakdown, a delayed acquisition is necessary.^4-6^ In context of the DIST-INFLAME substudy, a CTP with an adapted acquisition protocol will be performed prior to randomisation.

## 3.2. Imaging acquisition protocols DIST

The specific imaging protocols for acquisition of the NCCT, neuronavigation NCCT, CTA and CTP vary by centre. To allow for a structured systematic analysis of all image data and an automated imaging biomarkers extraction, standardized image acquisition protocols are important. Therefore, we describe the minimum requirements for image acquisition protocols to be used in the medical centres that participate in the DIST.

### 3.2.1. Non-contrast CT brain acquisition protocol requirements

**Non-contrast CT brain -** Version 1.0 February 1^st^ 2022

| Data acquisition | Parameters | | | Remarks |
| --- | --- | --- | --- | --- |
| Scan range | below foramen magnum - cranial vertex (includes entire sagittal sinus) | | | **obligatory** |
| Scan type | spiral **with** gantry/head tilt in orbitomeatal line | | | 1^st^ choice |
|  | spiral **without** gantry/head tilt in orbitomeatal line | | | 2^nd^ choice |
|  | sequential **with** gantry/head tilt in orbitomeatal line | | | 3^rd^ choice |
| Collimation | number of detector rows available × ≤ 1.0 mm | | | **preferred** |
| Rotation time (n.a. for sequential scanning) | cooperative patient | | ≥ 1.0 second | **preferred** |
|  | uncooperative (moving) patient | | ≤ 0.4 seconds | **preferred** |
| Pitch (n.a. for sequential scanning) | uncooperative (moving) patient | | 0.6-0.85 | **preferred** |
|  | moving patient | | 1.2-1.7 | **preferred** |
| Tube voltage (kVp) | local practice | | |  |
| Tube amperage (mAs) | local practice | | |  |
| CTDI_vol_ 16cm indication | 30-50 mGy (iterative) | | | 1^st^ choice |
|  | 50-70 mGy (filtered back projection) | | | 2^nd^ choice |
| Image reconstruction | Parameters | | | Remarks |
| Field of view | fit to skull | | | **obligatory** |
| Scan direction | caudal-cranial | | | **preferred** |
| Scan plane | axial | | | **obligatory** |
| Reconstructed slice thickness/increment | 1: | range 3-5 mm / 2.0-3.0 mm | | **obligatory** |
|  | 2: | ≤ 1.0 mm / ≤ 0.7 mm | | **obligatory** |
| Brain kernel | local practice | | |  |

n.a. = not applicable

### 3.2.2. CT-angiography head(-neck) acquisition protocol requirements

**CTA head(-neck) -** Version 1.1 December 22^nd^ 2022

| Data acquisition | Parameters | | | | | Remarks |
| --- | --- | --- | --- | --- | --- | --- |
| Scan range | cranial vertex (includes entire sagittal sinus) - below foramen magnum | | | | | **obligatory** |
|  | in case of CTA neck: just below aortic arch | | | | | **preferred** |
| Scan direction | cranial - caudal | | | | | **preferred** |
| Scan type | spiral | | | | | **preferred** |
| Collimation | number of detector rows available × ≤ 1.0 mm | | | | | **preferred** |
| Rotation time | cooperative patient | | | | ≥ 0.5 seconds | **preferred** |
|  | uncooperative (moving) patient | | | | ≤ 0.4 seconds | **preferred** |
| Pitch | cooperative patient | | | | 0.8-0.9 | **preferred** |
|  | uncooperative (moving) patient | | | | 1.2-1.7 | **preferred** |
| Tube voltage (kVp) | automated tube current selection for vascular exam type | | | | | 1^st^ choice |
|  | fixed kVp, as close to 100 kVp as possible | | | | | 2^nd^ choice |
| Tube amperage (mAs) | local practice | | | | |  |
| CTDI_vol_ indication (prior to kV/mA modulation) | 16 cm | 12-26 mGy (iterative) | | | | 1^st^ choice |
|  |  | 16-32 mGy (filtered back projection) | | | | 2^nd^ choice |
|  | 32 cm | 6-13 mGy (iterative) | | | | 1^st^ choice |
|  |  | 8-16 mGy (filtered back projection) | | | | 2^nd^ choice |
| Contrast media | flux (administered iodine in grams / second) | | | 1.3-1.8 * | | **obligatory** |
|  | maximum amount | | | 90 mL | | **obligatory** |
|  | injection site | | | right cubital fossa | | **preferred** |
| NaCl flush bolus amount | ≥ 40 mL | | | | | **obligatory** |
| Scan delay | timed with contrast bolus tracking | | | | | **obligatory** |
| Image reconstruction | Parameters | | | | | Remarks |
| Directions | axial | | | | | **obligatory** |
| Brain kernel | local practice | | | | |  |
| Reconstruction | Slice width/increment | | Field of view | | | Remarks |
| 1. Extracranial arteries (in case of CTA neck) | ≤ 1.0 mm / ≤ 0.6 mm | | small to fit carotids and vertebral arteries | | | **obligatory** |
| 2. Intracranial arteries | ≤ 0.75 mm / ≤ 0.4 mm | | small to fit intracranial arteries | | | **obligatory** |

* **Example contrast injection**:

Contrast media Visipaque: 320mg iodine / mL = 0.320 g iodine / mL.

Example calculation flowrate at flux 1.3 is: 1.3 / 0.320 = 4.0 mL / second.

**Considerations**:

A higher iodine flux is preferred over a lower iodine flux, but it should be feasible over intravenous canula in the individual patient.

With faster scans/scanners injection protocol tends to shift to lower volumes due to shorter scan time, but a resulting drop in peak HU needs to be compensated with higher flux.

Low kVp (< 100) can tolerate lower iodine flux compared to high kVp (≥ 100) scans.

### 3.3.3. CT-perfusion brain acquisition protocol requirements

**CTP brain -** Version 1.1 August 29^th^ 2023

| Data acquisition | Parameters | | | | Remarks |
| --- | --- | --- | --- | --- | --- |
| Moment of acquisition | before CTA or >15 min after CTA | | | | **obligatory** |
| Tube voltage (kVp) | local practice | | | |  |
| Tube amperage (mAs) | local practice | | | |  |
| Start of acquisition | delay < 6 seconds | | | | **obligatory** |
|  | direct with as little delay as possible | | | | **preferred** |
| Max acquisition time | 210 seconds | | | | **obligatory** |
| Acquisition sequence | first 60 s according to preferred acquisition sequence or local practice + 5 x 30 s | | | | **obligatory** |
|  | Canon Aquilion | | 20 x 2 s + 4 x 5 s + 5 x 30 s | | **preferred** |
|  | Siemens Somatom | | 20 x 1.5 s + 10 x 3 s + 5 x 30 s | | **preferred** |
|  | Philips Brilliance | | 30 x 2 s + 5 x 30 s | | **preferred** |
|  | Philips iQON | | 18 x 3.4 s + 5 x 30 s | | **preferred** |
| Contrast media | flux (administered iodine in grams / second) | | | 1.8 * | **obligatory** |
|  | total iodine dose | | | 15 g * | **obligatory** |
|  | injection site | | | right cubital fossa | **preferred** |
| NaCl flush bolus amount | 40 mL | | | | **obligatory** |
| Image reconstruction | Parameters | | | | Remarks |
| Field of view | whole brain | | | | **obligatory** |
| Brain coverage | ≥80 mm | | | | **obligatory** |
|  | whole brain | | | | **preferred** |
| Reconstructed slice thickness/increment | 1: | 5 mm / ≤ 3.0 mm | | | **obligatory** |
|  | 2: | ≤ 1.5 mm / ≤ 1.0 mm | | | **obligatory** |

| *** Corresponding contrast volume and injection rate per iodine concentration used** | | |
| --- | --- | --- |
| Iodine concentration | Contrast volume | Injection rate |
| 270 mg/ml | 55.6 ml | 6.7 ml/s |
| 300 mg/ml | 50 ml | 6 ml/s |
| 320 mg/ml | 46.9 ml | 5.6 ml/s |
| 350 mg/ml | 42.9 ml | 5.1 ml/s |
| 400 mg/ml | 37.5 ml | 4.5 ml/s |

# 4. References

1. Vespa P, Hanley D, Betz J, et al. ICES (Intraoperative Stereotactic Computed Tomography-Guided Endoscopic Surgery) for brain hemorrhage: a multicenter randomized controlled trial. *Stroke*. Nov 2016;47(11):2749-2755. doi:10.1161/STROKEAHA.116.013837

2. Kellner CP, Chartrain AG, Nistal DA, et al. The Stereotactic Intracerebral Hemorrhage Underwater Blood Aspiration (SCUBA) technique for minimally invasive endoscopic intracerebral hemorrhage evacuation. *J Neurointerv Surg*. Aug 2018;10(8):771-776. doi:10.1136/neurintsurg-2017-013719

3. Rothrock RJ, Chartrain AG, Scaggiante J, et al. Advanced techniques for endoscopic intracerebral hemorrhage evacuation: a technical report with case examples. *Oper Neurosurg (Hagerstown)*. Dec 15 2020;20(1):119-129. doi:10.1093/ons/opaa089

4. Dankbaar JW, Hom J, Schneider T, et al. Dynamic perfusion CT assessment of the blood-brain barrier permeability: first pass versus delayed acquisition. *AJNR Am J Neuroradiol*. Oct 2008;29(9):1671-6. doi:10.3174/ajnr.A1203

5. Hom J, Dankbaar JW, Schneider T, Cheng SC, Bredno J, Wintermark M. Optimal duration of acquisition for dynamic perfusion CT assessment of blood-brain barrier permeability using the Patlak model. *AJNR Am J Neuroradiol*. Aug 2009;30(7):1366-70. doi:10.3174/ajnr.A1592

6. Bennink E, Riordan AJ, Horsch AD, Dankbaar JW, Velthuis BK, de Jong HW. A fast nonlinear regression method for estimating permeability in CT perfusion imaging. *J Cereb Blood Flow Metab*. Nov 2013;33(11):1743-51. doi:10.1038/jcbfm.2013.122
